# Discovery of Novel MicroRNAs in Rat Kidney Using Next Generation Sequencing and Microarray Validation

**DOI:** 10.1371/journal.pone.0034394

**Published:** 2012-03-28

**Authors:** Fanxue Meng, Michael Hackenberg, Zhiguang Li, Jian Yan, Tao Chen

**Affiliations:** 1 Division of Genetic and Molecular Toxicology, National Center for Toxicological Research, Food and Drug Administration, Jefferson, Arkansas, United States of America; 2 Dpto. de Genética, Facultad de Ciencias, Universidad de Granada, Granada, Spain; University of Illinois-Chicago, United States of America

## Abstract

MicroRNAs (miRNAs) are small non-coding RNAs that regulate a variety of biological processes. The latest version of the miRBase database (Release 18) includes 1,157 mouse and 680 rat mature miRNAs. Only one new rat mature miRNA was added to the rat miRNA database from version 16 to version 18 of miRBase, suggesting that many rat miRNAs remain to be discovered. Given the importance of rat as a model organism, discovery of the completed set of rat miRNAs is necessary for understanding rat miRNA regulation. In this study, next generation sequencing (NGS), microarray analysis and bioinformatics technologies were applied to discover novel miRNAs in rat kidneys. MiRanalyzer was utilized to analyze the sequences of the small RNAs generated from NGS analysis of rat kidney samples. Hundreds of novel miRNA candidates were examined according to the mappings of their reads to the rat genome, presence of sequences that can form a miRNA hairpin structure around the mapped locations, Dicer cleavage patterns, and the levels of their expression determined by both NGS and microarray analyses. Nine novel rat hairpin precursor miRNAs (pre-miRNA) were discovered with high confidence. Five of the novel pre-miRNAs are also reported in other species while four of them are rat specific. In summary, 9 novel pre-miRNAs (14 novel mature miRNAs) were identified via combination of NGS, microarray and bioinformatics high-throughput technologies.

## Introduction

MicroRNAs (miRNAs) are small non-coding RNAs of ∼22 nucleotides in length and ubiquitously present in plant and animal cells [1]. miRNAs play an important role in the post-transcriptional regulation of gene expression via binding to the 3′ UTR region of the target mRNAs, resulting in mRNA degradation or translation inhibition [2]. Recent studies indicate that miRNAs are critical for many physiological processes, including cell proliferation, cell differentiation, and cell death [3], [4]. Dysregulated miRNAs have been found in different types of human diseases and tumors [5], [6], [7].

miRNA genes are initially transcribed by RNA polymerase II to generate primary miRNAs (pri-miRNAs). Pri-miRNAs are processed by RNase Drosha to release approximately 70 nucleotides long miRNA precursors (pre-miRNAs) that have characteristic hairpin structures. Pre-miRNAs are then exported from the nucleus to the cytoplasm. RNase Dicer cleaves the pre-miRNA hairpin to generate a double-stranded miRNA duplex with a characteristic 3′ 2-nucleotide overhang. Subsequently, the double-stranded miRNA duplex is separated and one strand is selected as the mature miRNA, whereas most of the other strand that are named as mature* sequences is degraded [8], [9]. Sometimes, mature variants generated from the same miRNA precursor contain different sequences from the mature and/or mature* sequence. These mature variants are named as isomirs [10]. The characteristic structures of these different stages of miRNA biogenesis, such as hairpin structures and mature* sequences, have been utilized for identification of novel miRNAs based on certain guidelines [11], [12]. The criteria for decision of novel miRNAs depend on whether the novel miRNAs have homologous ones in other species. Due to the phylogenetic conservation of miRNAs, the requirements for defining homologous miRNAs are generally less strict than those for species-specific miRNAs such as those found in rats only [11], [12].

The first two miRNAs, *lin-4 and let-7*, were discovered in the *Caenorhabditis elegans* [13], [14]. Subsequently, about 100 miRNAs were identified by cloning and Sanger sequencing [15], [16], [17], [18], [19]. However, such approaches were limited in their ability to detect rare miRNAs, or tissue-specific miRNAs from tissues that are difficult to obtain. Next generation sequencing (NGS), a high-throughput technology, has dramatically changed the nature of biomedical research and medicine since 2005. NGS is a combination of various procedures that includes template preparation, sequencing and imaging, and genome alignment and assembly. This new technology markedly reduces the cost and time required to sequence large amounts of DNA [20], [21], [22], [23], [24]. Also, unlike PCR- or microarray-based sequencing technologies, NGS can easily recognize unknown DNA sequences. Thus, NGS can be used to identify new gene sequences. Previous studies showed that NGS can successfully discover low abundance novel miRNAs in different species by reverse-transcription of miRNAs to their cDNAs [25], [26], [27].

Since NGS platforms can generate several gigabases of sequencing data per run, bioinformatics tools are required to process the huge amount of data. Several tools have been widely used for miRNA transcriptomic analysis of NGS data to discover novel miRNAs, including miRDeep [28], [29], [30], [31], miRDeep2 [32], miRDeep-p [33], miRanalyzer [34], [35], [36], miRExpress [37], deepBase [38], miRTRAP [39], mirTools [40], SSCprofilter [41], [42], mirExplorer [43], and MIReNA [44]. Although these tools use different algorithms to predict novel miRNAs, they share the same two basic principles: 1) mapping of the reads to the genome and 2) checking for the presence of a hairpin structure in the genome. In addition, existence of mature* sequence and a Dicer cleavage pattern provide further evidence for a miRNA. In this study, the miRanalyzer standalone version was utilized for the discovery of novel rat miRNAs.

Currently, there are three common methods for measuring miRNAs, microarrays, quantitative PCR (qPCR) and NGS. The clear advantage of NGS over microarrays and qPCR is its capability for identification of novel miRNAs because microarrays and qPCR detect miRNAs based on known miRNA sequences. However, different steps of NGS, such as template preparation, RNA ligation, PCR amplification and imaging, can introduce errors. Therefore, novel miRNAs discovered by NGS need to be validated through other platforms. Although qPCR is often considered a “gold standard” in the detection and quantization of gene expression, it is not a high-throughput application for miRNA expression. According to our previous study, expression of miRNAs measured by TaqMan quantitative real-time PCR is comparable with that of LC Sciences' microarray analysis [45]. Microarrays are still the best choice for high-throughput analysis of miRNA expression. Microarrays and NGS can be used for mutual validation of miRNA expression [46].

Currently, 21,643 mature miRNAs have been discovered and deposited in the publically available miRNA database, miRBase (version 18.0, November 2011; http://miRNA.sanger.ac.uk/sequences/index.shtml). The database contains 1,921 miRNAs from human, 1,157 from mouse and 680 from rat. Despite the importance of the rat as a model organism, the number of known rat miRNAs is not comparable to those for human and mouse, considering the conserved nature of miRNAs among different species. Therefore, it is very important to discover the unknown rat miRNAs and explore their functional roles. In this study, NGS was employed to sequence small RNAs in rat kidney; miRanalyzer was applied for identifying known and unknown rat miRNAs; and a custom vertebrate miRNA array containing more than five thousand known vertebrate miRNAs and a hundred novel rat miRNA candidates determined by the NGS analysis was designed to verify novel rat miRNAs. These two high throughput technologies, in combination with a potent tool for miRNA bioinformatics and biostatistics analyses, helped us discover 9 novel rat pre-miRNAs, which express 14 novel mature miRNA sequences.

## Results

### Recognition of Rat Homologous Novel miRNAs

Small RNA transcriptomes of kidney samples from 8 rats, 4 treated with aristolochic acid (AA) and 4 untreated as control, were analyzed using NGS (NGS data are available through Gene Expression Omnibus series accession numbers GSE33703). AA is Group 1 carcinogen and able to induce the rat kidney tumors. Our previous study (manuscript is in preparation) showed that many miRNAs expressions increased in the AA treatment group, compared with those of in control group. Using samples from different animals as well as AA-treated and untreated rats should strengthen the discovery of novel miRNAs as accidental discovery due to fluctuations can be virtually discarded.

**Figure 1:**
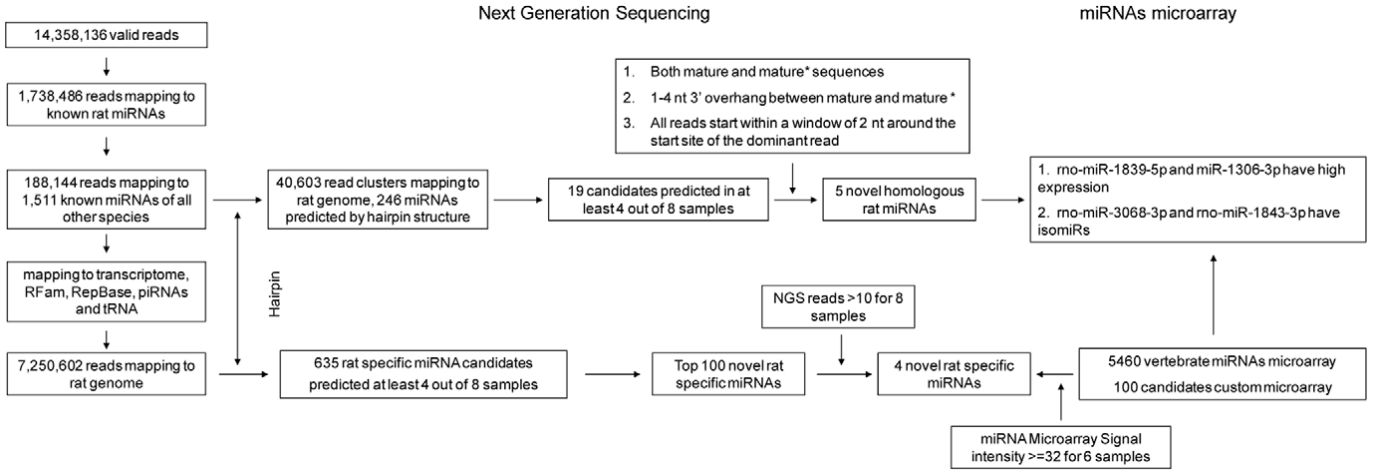
Scheme of the work flow for identifying novel rat miRNAs.

The sequencing data were input into miRanalyzer, a web server and stand-alone tool, to predict both novel homologous and rat-specific miRNAs [34]. A schema of the sequence analysis workflow is shown in Figure 1. The tool first removed all reads with ‘N’ (or other ambiguous bases) and those shorter than 17 bases. Reads longer than 26 bases were trimmed and regrouped, because the bases of miRNAs are normally ranging from 17 to 25. In total, 14,358,136 reads were obtained from the 8 rat kidney samples.

**Figure 2:**
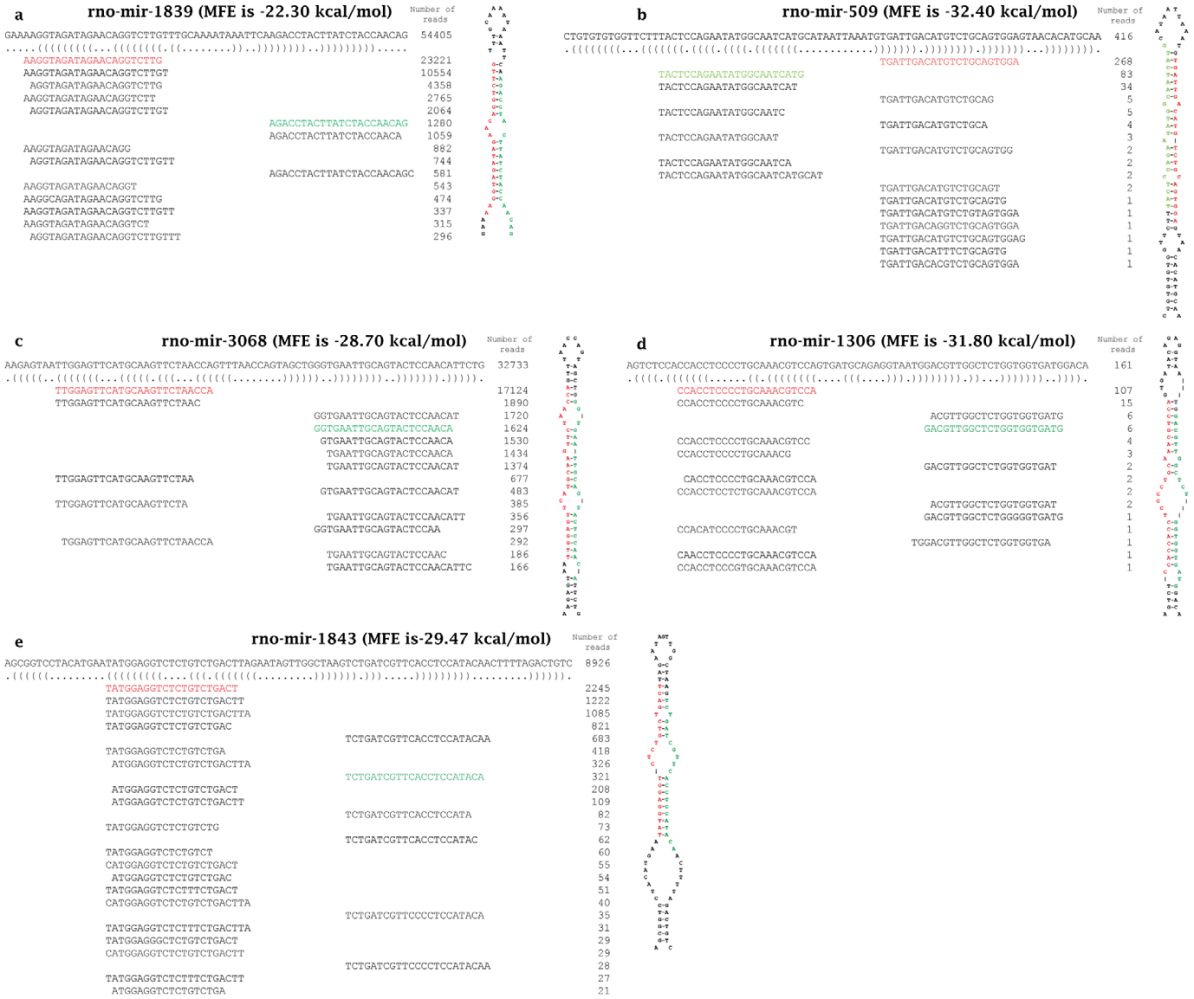
The sequences and secondary structures of five novel rat pre-miRNAs homologous to known miRNAs in other species. 2a. rno-mir-1839. 2b. rno-mir-509. 2c. rno-mir-3068. 2d. rno-mir-1306. 2e. rno-mir-1843. The sequences of 5 novel rat homologous pre-miRNAs hairpin are depicted above their dot-bracket notation secondary structures as determined by RNAfold [62], [63] using minimum free energy algorithm (MFE). RNAfold is a widely used webserver to predict RNA secondary structure. Below the dot-bracket notation secondary structures of these novel rat homologous pre-miRNAs, each of the small RNA sequences that matched those pre-miRNAs hairpin are listed, with the number of reads representing each sequence at its right side. The mature and the mature* sequences are marked in red and green respectively. The MFEs of those rat novel pre-miRNAs predicted by RNAfold are above their sequences. The single nucleotide extension isomirs of the mature* sequences had higher read counts than the mature* sequences with perfect 2 nt 3′ overhang in two miRNAs (rno-miR-3068-3p and rno-miR-1843-3p).

Rat homologous novel miRNAs are those miRNAs that have been reported in other species but not in rat. To find the rat homologous novel miRNAs, the known rat miRNAs were first removed. There were 1,738,486 reads that were mapped to known rat miRNAs and were eliminated from further analysis. The remaining reads were then aligned to a non-redundant set of known mature miRNAs from all other species (miRBase version 17), yielding 188,144 mapped reads. In total, 1,511 miRNAs were detected by at least one read in at least 1 out of the 8 sequencing samples. After mapping those reads to the genome, 40,603 read clusters considered as putative mature miRNA sequences were acquired. Genome sequences around the position of the read cluster were extracted and the energetically best hairpin structures were retained as putative pre-miRNAs if they had (i) at least 19 base pairings in the secondary structure and (ii) at least 11 base pairings located in the read cluster region (number of pairings between putative mature and mature*). After applying the minimum number of base pairings to the 40,603 pre-microRNA candidates (one for each read cluster) and forcing a hairpin secondary structure we obtain 13,336 candidates that are used as input for the machine learning prediction. Eventually 246 putative novel miRNAs were predicted in the 8 samples. After comparing the information across the 8 samples by using the differential expression module of miRanalyzer, 19 pre-miRNA candidates were predicted in at least 4 out of the 8 samples. After realigning the reads to the consensus sequences of these 19 pre-miRNA candidates, the cleavage pattern was analyzed. The homologous pre-miRNAs were considered as novel pre-miRNAs if they had (i) both the mature and mature* sequences, (ii) a characteristic 1–4 nt 3′ overhang between mature and mature* sequences, and (iii) less than 2 nt fluctuation of read start sites around the start site of the predominant read (the read with the highest expression value). After applying these structural criteria, 5 novel pre-miRNAs homologous to known miRNAs in other species were discovered and named as rno-mir-1839, rno-mir-3068, rno-mir-1843, rno-mir-509 and rno-mir-1306. As mature and mature* sequences are derived from the opposite arms of the hairpin pre-miRNAs, they were named novel rat homologous miRNAs according to their locations. For example, rno-miR-1839-5p and rno-miR-1839-3p were named because the two miRNAs were considered as the 5′ and 3′ arms of rno-mir-1839 pre-miRNA. Therefore, 10 rat homologous miRNAs were generated from 5 rat homologous pre-miRNAs. Although these 5 pre-miRNAs have been detected in mouse, they have not been previously reported in rat. All these 10 rat homologous miRNAs possess a perfect 2 nt 3′ overhang that is a consequence of the Dicer cleavage. In addition, these novel miRNAs were detected in multiple samples (at least 4 out of the 8 samples) with reads >10 (except rno-miR-1306-3p) [42]. Therefore, all of the 10 novel miRNAs are high-confidence novel rat homologous miRNAs according to the guidelines for novel miRNAs. The sequences and the secondary structures of these 5 novel rat homologous pre-miRNAs are shown in Figure 2. The single nucleotide extension isomirs of the mature* sequences had higher read counts than the mature* sequences in two miRNAs (rno-miR-3068-3p and rno-miR-1843-3p) (Figure 2c and 2e). Table 1 shows sequences and genome locations of 10 rat homologous miRNAs. All Sequences of novel rat miRNAs are the same as those of other species except rno-miR-3068-5p, rno-miR-509-3p and rno-miR-1306-3p. Table 2 shows homologous miRNAs and the homologous sequences of the 10 novel rat homologous miRNAs. All novel rat miRNAs have homologous sequences in mouse except rno-miR-1306-5p and rno-miR-509-3. Table 3 shows NGS read counts and microarray signal intensities of the 10 novel rat homologous miRNAs, which were used in the miRNA identification and validation in this study. Table 4 shows sequences of the 5 novel rat homologous pre-miRNAs.

**Table 1:** Ten rat homologous miRNAs sequences and genome locations.

Novel Rat miRNA	Mature Sequence	Chromosome	Start – End	Strand
**rno-miR-1839-5p**	AAGGUAGAUAGAACAGGUCUUG	1	137744048–137744110	+
rno-miR-1839-3p	AGACCUACUUAUCUACCAACAG			
**rno-miR-3068-5p**	UUGGAGUUCAUGCAAGUUCUAACC*A*	6	111674240–111674314	−
rno-miR-3068-3p	GGUGAAUUGCAGUACUCCAACA (#)			
**rno-miR-1843-5p**	UAUGGAGGUCUCUGUCUGACU	6	103413903–103413991	−
rno-miR-1843-3p	UCUGAUCGUUCACCUCCAUACA (#)			
**rno-miR-509-5p**	UACUCCAGAAUAUGGCAAUCAUG	X	154190989–154191072	−
rno-miR-509-3p	UGAUUGACA***U***GUCUGCAG*UGGA*			
**rno-miR-1306-5p**	CCACCUCCCCUGCAAACGUCCA	11	84703718–84703789	+
rno-miR-1306-3p	*G*ACGUUGGCUCUGGUGGUGAU*G*			

Note: The names of novel rat mature and mature* miRNAs are marked in bold and regular font, respectively. Sequences of three novel rat miRNAs differ from homologous sequences related to other species and those different bases are marked in italic font. All miRNAs show a perfect 2 nt 3′ overhang, except rno-miR-3068-3p and rno-miR-1843-3p marked with (#). Those two miRNAs have perfect Dicer pattern with the second most expressed mature* read, while the most expressed mature* read probably is a single nucleotide extension isomiR.

**Table 2:** Homologous miRNAs and homologous sequences related to ten novel rat homologous miRNAs.

Novel Rat miRNA	Homologous miRNA	Homologous Sequence
**rno-miR-1839-5p**	cfa-miR-1839#mmu-miR-1839-5p#bta-miR-1839#eca-miR-1839#ssc-miR-1839-5p	AAGGUAGAUAGAACAGGUCUUG
rno-miR-1839-3p	mmu-miR-1839-3p	AGACCUACUUAUCUACCAACAG
**rno-miR-3068-5p**	mmu-miR-3068	UUGGAGUUCAUGCAAGUUCUAACC
rno-miR-3068-3p	mmu-miR-3068*	GGUGAAUUGCAGUACUCCAACA
**rno-miR-1843-5p**	mmu-miR-1843-5p	UAUGGAGGUCUCUGUCUGACU
rno-miR-1843-3p	mmu-miR-1843-3p	UCUGAUCGUUCACCUCCAUACA
**rno-miR-509-5p**	mmu-miR-509-5p	UACUCCAGAAUGUGGCAAUCAU
rno-miR-509-3p	age-miR-509b	UGAUUGACACGUCUGCAGAUAGA
	age-miR-509a	UGAUUGACACGUCUGCAGGUAGA
**rno-miR-1306-5p**	ssc-miR-1306-5p	CCACCUCCCCUGCAAACGUCCA
rno-miR-1306-3p	mmu-miR-1306-3p#ssc-miR-1306-3p	ACGUUGGCUCUGGUGGUGAU

**Table 3:** Ten novel rat homologous miRNAs - NGS read counts and microarray signal intensities.

Novel Rat miRNA	NGS Reads								miRNA Array Signal Intensity					
	CTL13	CTL14	CTL15	CTL16	AA19	AA20	AA21	AA22	CTL13	CTL14	CTL15	AA19	AA20	AA21
rno-miR-1839-5p	8162	7238	8496	5294	6110	6872	5256	5137	85.42	92.51	82.01	63.97	129.1	37.79
rno-miR-1839-3p	378	389	389	462	409	474	402	482	9.28	18.79	8.19	6.79	27.95	2.46
rno-miR-3068-5p	2288	2212	2190	3636	2506	1935	1884	5459	14.85	12.03	16.36	44.82	7.27	27.57
rno-miR-3068-3p	1477	1593	1775	1208	1285	1270	1169	1128	5.35	16.6	22.47	25.82	7.16	30.01
rno-miR-1843-5p	1181	970	1063	634	745	806	848	715	11.81	6.07	6.87	11.23	9.49	11.4
rno-miR-1843-3p	165	181	172	148	101	239	206	167	4.13	11.73	14.4	13.19	6.97	9.04
rno-miR-509-5p	9	16	30	10	8	8	36	12	0.67	0.03	1.11	0.02	0	3.19
rno-miR-509-3p	53	34	46	23	11	15	52	47	0	2.58	0.01	0.03	0	1.92
rno-miR-1306-5p	11	13	4	10	12	21	17	28	0	6.71	0.02	20.73	0	1.94
rno-miR-1306-3p	0	0	1	1	0	0	0	4	94.92	89.46	53.45	24.31	42.25	130.4

Note: rat kidney samples in the control group: CTL13, CTL14, CTL15 and CTL16; rat kidney samples in the AA treated group: AA19, AA20, AA21, and AA22.

**Table 4:** Five novel rat homologous pre-miRNAs sequences.

Novel Rat pre-miRNA	Precursor Sequence
rno-mir-1839	GAAAAGGUAGAUAGAACAGGUCUUGUUUGCAAAAUAAAUUCAAGACCUACUUAUCUACCAACAG
rno-mir-3068	AAGAGUAAUUGGAGUUCAUGCAAGUUCUAACCAGUUUAACCAGUAGCUGGGUGAAUUGCAGUACUCCAACAUUCUG
rno-mir-1843	AGCGGUCCUACAUGAAUAUGGAGGUCUCUGUCUGACUUAGAAUAGUUGGCUAAGUCUGAUCGUUCACCUCCAUACAACUUUUAGACUGUC
rno-mir-509	CUGUGUGUGGUUCUUUACUCCAGAAUAUGGCAAUCAUGCAUAAUUAAAUGUGAUUGACAUGUCUGCAGUGGAGUAACACAUGCAA
rno-mir-1306	AGUCUCCACCACCUCCCCUGCAAACGUCCAGUGAUGCAGAGGUAAUGGACGUUGGCUCUGGUGGUGAUGGACA

### Recognition of Rat-Specific Novel miRNAs

For detection of the rat-specific novel miRNAs, all reads that were mapped to known miRNAs, transcriptome, RFam, RepBase, piRNAs and tRNA were removed first. Of the remaining reads, 7,250,602 could be mapped to the rat genome and were used for the prediction of the novel miRNAs. The predictions were performed as described previously [34] and resulted in 635 novel miRNAs candidates. These candidates were expressed in at least 4 of the 8 samples (default settings of miRanalyzer). Although these miRNA candidates are rat-specific in the sense that they have not been detected in any other species, it does not rule out that they might exist in other species as well.

### Validation of the Novel miRNAs

To validate these rat homologous miRNAs and rat-specific miRNA candidates, custom vertebrate miRNA microarray (microarray data are available through Gene Expression Omnibus series accession numbers GSE33360) was performed in 3 untreated and 3 AA treated rat kidney samples which were also used in the NGS analysis. Vertebrate miRNA array from IC Sciences covers all 5,460 miRNAs from 32 vertebrates based on miRBase version 17. In addition, the complementary probes to the mature sequences of the top 100 of 635 novel rat-specific candidates generated via the NGS analysis were added to the miRNA array (100 custom probes are the limit of custom miRNA microarray made by LC sciences). Thus, the expression levels of a total of 5,560 miRNAs were measured using this high throughput platform. Since miRNA genes tend to be conserved across species, the 5,460s vertebrate miRNAs could be used to validate the expression of novel rat homologous miRNAs. At the same time, the 100 rat-specific miRNA probes in the array could be used to validate the expression of these miRNA candidates resulted from the NGS analysis. The microarray data showed that 1,495 out of 5,560 miRNAs were expressed at different levels when microarray signal intensity cutoff was set to 32 for determination of miRNA expression as the manufacturer's suggestion.

Two novel homologous miRNAs (rno-miR-1839-5p, rno-miR-1306-3p) meet the manufacturer's (LC Sciences) criteria and further support they are novel rat miRNAs. rno-miR-1839-5p had consistent NGS read counts of more than 1000 in all of the 8 NGS samples and was significantly expressed in all 6 samples as determined by the microarray analysis. Although rno-miR-1306-3p had very low read counts (NGS reads counts are between 1 and 4 in 3 of 8 samples), it was consistently expressed in 5 of 6 samples as determined by the microarray analysis. Therefore, rno-miR-1306-3p is qualified as a novel rat miRNA [11].

**Figure 3:**
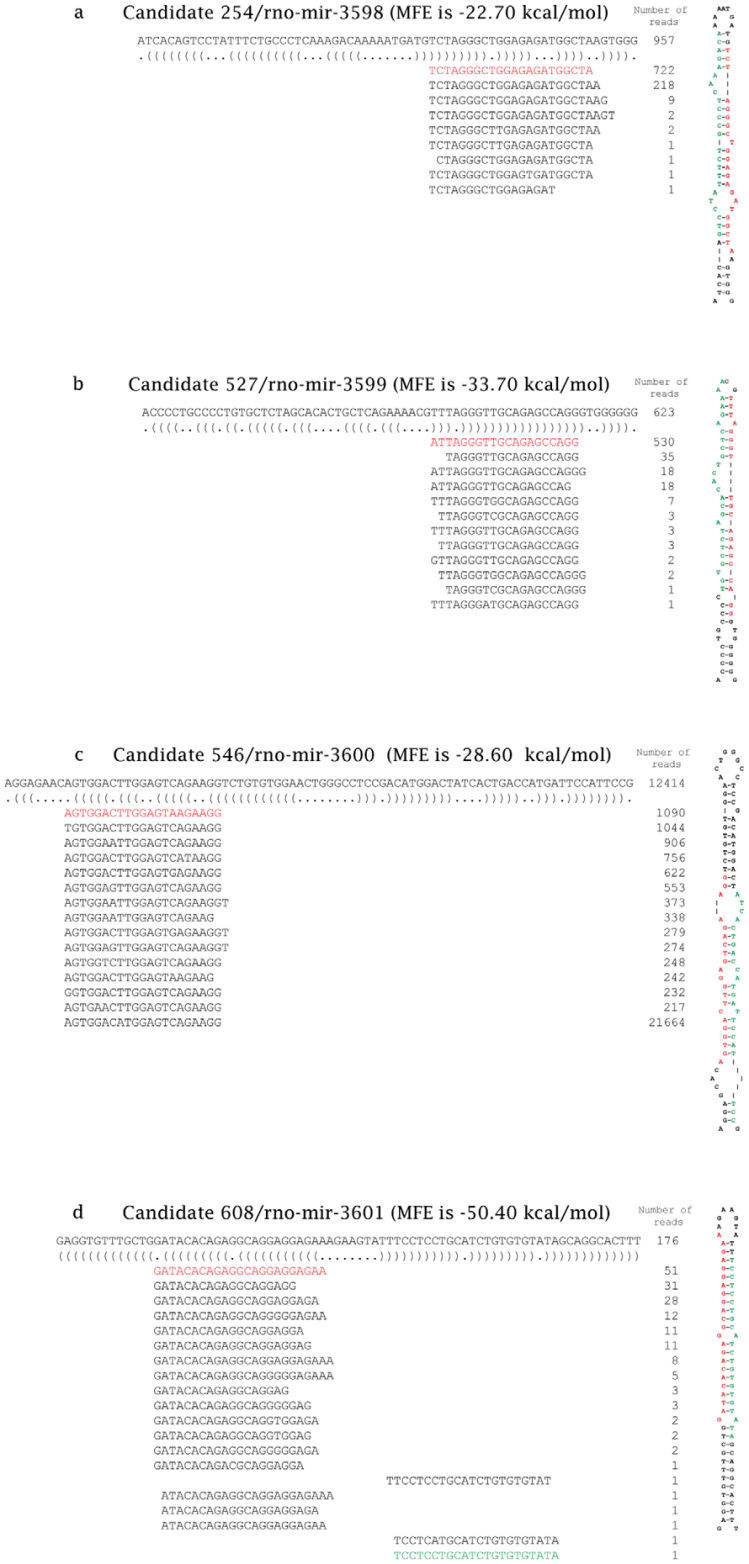
The sequences and secondary structures of the four novel rat specific pre-miRNAs. 3a. rno-mir-3598. 3b. rno-mir-3599. 3c. rno-mir-3600. 3d. rno-mir-3601. The sequences of 4 novel rat specific pre-miRNAs are depicted above their dot-bracket notation secondary structures as determined by RNAfold [62], [63] using MFE. RNAfold is a widely used webserver to predict RNA secondary structure. Below the dot-bracket notation secondary structures of these rat specific pre-miRNA, each of the small RNA sequences that matched those pre-miRNAs hairpin are listed, with the number of reads representing each sequence at its right side. The mature and the mature* sequences are marked in red and green, respectively. The MFEs of those rat specific miRNAs predicted by RNAfold are above their pre-miRNA sequences. For rno-miR-3598, the inferred mature* sequence is shown in green in the secondary structure.

Strict criteria were applied to define the rat-specific novel miRNA candidates. The cutoff for NGS read count was set to 10 [12] and that for miRNA array signal intensity was set to 32 for every sample [47]. Six rat-specific novel miRNA candidates were matched to these criteria. After realigning their sequences to the pre-miRNA sequences, two of the six candidates were discarded due to high fluctuations of the read start positions. The remaining four novel miRNA candidates were considered as novel rat-specific miRNAs. They were named as rno-miR-3598, rno-miR-3599, rno-miR-3600 and rno-miR-3601, respectively. The alignments and secondary structures for these novel miRNAs are displayed in Figures 3. Their mature sequences and genome positions, NGS read counts and microarray signal intensities, and precursor sequences are shown in Tables 5, 6 and 7, respectively. For rno-miR-3601, the mature* sequence was also detected in the NGS analysis and its sequence alignment and hairpin structure are shown in Figure 3d. Also, the expression level of rno-miR-3598 was significantly altered by the treatment of AA according to the microarray analysis (*P* = 0.0134).

**Table 5:** Four rat-specific novel miRNAs - Mature sequences and genome locations.

Novel Rat miRNA	Mature Sequence	Chromosome	Start - End	Strand
rno-miR-3598	UCUAGGGCUGGAGAGAUGGCUA	13	40705693–40705785	+
rno-miR-3599	AUUAGGGUUGCAGAGCCAGG	5	158395573–158395709	−
rno-miR-3600	UGUGGACUUGGAGUCAGAAGG	5	5156340–5156444	−
rno-miR-3601	GAUACACAGAGGCAGGAGGAGAA	3	41992498–41992610	−

**Table 6:** Four rat-specific novel miRNAs - NGS read counts and microarray signal intensities.

Novel Rat miRNA	NGS Reads								miRNA Array Signal Intensity					
	CTL13	CTL14	CTL15	CTL16	AA19	AA20	AA21	AA22	CTL13	CTL14	CTL15	AA19	AA20	AA21
rno-miR-3598	80	88	81	80	150	109	80	289	164	173	180	312	315	391
rno-miR-3599	90	70	129	67	43	89	84	51	134	173	74	73	52	166
rno-miR-3600	3058	2741	1838	343	1095	1152	1595	592	316	236	223	164	133	254
rno-miR-3601	17	15	34	11	25	28	28	15	42968	33213	39003	47376	25834	48927

Note: rat kidney samples in the control group: CTL13, CTL14, CTL15, and CTL16; rat kidney samples in the AA treated group: AA19, AA20, AA21, and AA22.

**Table 7:** Four rat-specific novel pre-miRNAs sequences.

Novel Rat pre-miRNA	Precursor Sequence
rno-mir-3598	AUCACAGUCCUAUUUCUGCCCUCAAAGACAAAAAUGAUGUCUAGGGCUGGAGAGAUGGCUAAGUGGG
rno-mir-3599	ACCCCUGCCCCUGUGCUCUAGCACACUGCUCAGAAAACGUUUAGGGUUGCAGAGCCAGGGUGGGGGG
rno-mir-3600	AGGAGAACAGUGGACUUGGAGUCAGAAGGUCUGUGUGGAACUGGGCCUCCGACAUGGACUAUCACUGACCAUGAUUCCAUUCCG
rno-mir-3601	GAGGUGUUUGCUGGAUACACAGAGGCAGGAGGAGAAAGAAGUAUUUCCUCCUGCAUCUGUGUGUAUAGCAGGCACUUU

### Predicted Targets of the Novel miRNAs

TargetSpy was chosen to predict the target genes of the 14 novel miRNAs by forcing the existence of a seed *in silico* [48], [49] In total, 6918 target genes were identified for future functional analysis (Data S1).

## Discussion

Currently, there are two guidelines for discovery of novel miRNAs, Ambros guideline and Griffiths-Jones guideline. The Ambros guideline is a general guideline [11], while the Griffiths-Jones guideline is a specific guideline for the discovery of novel miRNAs using NGS data [12]. Both guidelines contain expression and biogenesis criteria. In the Ambros guideline, expression criteria include detection of miRNAs by hybridization (such as northern blot, Taqman real time PCR or microarray) and cloning and Sanger sequencing. Biogenesis criteria include classic hairpin structure, phylogenetic conservation, and Dicer function. miRNAs must meet at least 1 expression criterion and 1 biogenesis criterion (although Dicer function only provides further evidence and it can not be used as an independent biogenesis criterion). In addition, the Ambros guidelines suggest that “very close homologs in other species can be annotated as miRNA orthologs without experimental validation, if they satisfy “the criterion of a high degree of phylogenetic conservation” [11]. In the Griffiths-Jones guideline, expression criterion is multiple reads from multiple independent experiments (cutoff is 10–20). Biogenesis criteria are reads being able to map to the genome, sequence flanking the putative mature miRNAs showing a hairpin structure, mapped reads without overlapping of other RNAs, conserved 5′-end of the mature sequence, and the existence of mature* sequence and correct 3′ overhang. The Griffiths-Jones guideline considers that consistent 5′-end processing and mature* sequences are critical for discrimination between high-confidence miRNAs and fragments of other RNAs in NGS data.

In our study, both guidelines were utilized to identify novel rat miRNAs. Ten rat novel homologous miRNAs meet all Griffiths-Jones criteria except rno-miR-1306-3p that was validated by the microarray analysis, and meets the Ambros criteria. Four rat-specific miRNAs meet at least 4 of 5 Griffiths-Jones criteria (rno-miR-3601 meets 5/5 criteria). In addition, they were confirmed by the microarray analysis. Thus, all four rat-specific miRNAs meet Ambros criteria too. Thus, all 14 miRNAs generated from 9 pre-miRNAs are high-confidence miRNAs according to the both guidelines.

NGS and microarrays are two high-throughput platforms for analysis of gene and miRNA expression. NGS is able to assess the copy number of transcripts and provides “digital gene expression” while microarrays measure relative gene expression. Although there are debates on accuracy and reliability of the two platforms [50], [51], [52], [53], they are generally considered as comparable and can be used for validation of each other [46], [54]. In this study, both NGS and microarray analyses were applied to identify and validate novel miRNAs in rat kidneys. Novel miRNAs that express in both the platforms are more reliable than those that only express in one platform. Therefore, 4 rat-specific miRNAs (rno-miR-3598, rno-miR-3599, rno-miR-3600, rno-miR-3601) and 2 rat homologous miRNAs (rno-miR-1839-5p and rno-miR-1306-3p) are high-confidence rat miRNAs. Although other 8 miRNAs and their isoforms (rno-miR-1839-3p, rno-miR-3068-5p, rno-miR-3068-3p, rno-miR-1843-5p, rno-miR-1843-3p, rno-miR-509-5p, rno-miR-509-3p, rno-miR-1306-5p) were not confirmed by microarray analysis, they are still considered as high-confidence novel miRNAs because they satisfy the Ambros guidelines [11]. Also, miRNA expression detected using NGS may not be able to be found by means of microarrays because the overlapping of expressed genes between NGS and microarray platforms is about 40–50% [54]. It may be due to NGS's high sensitivity in detecting the genes with low expression levels than microarrays [27]. Thus, the low level of expression of rno-miR-509-5p, rno-miR-509-3p, rno-miR-1306-5p and rno-miR-509-3p measured by NGS might not be detected by the microarray.

It is estimated that miRNAs target about 60% of protein-coding genes [55] and miRNAs play important roles in a variety of diseases and disorders [5], [7]. The potential miRNAs targets predicted by targetspy and their functions need to be further studied, Given that AA is a top 2 potent human carcinogen that induces kidney tumors in rats [56], rno-miR-3598 may be the potential used as a kidney tumor biomarker for AA exposure.

In summary, NGS, microarray gene expression analysis and bioinformatics tools were used for analysis of small RNA data generated from rat kidneys. These combined approaches resulted in discovery of 14 high confidence novel rat miRNAs based on Ambros and Griffiths-Jones guidelines. Ten novel miRNAs from 5 pre-miRNAs are homologues to other species while four miRNAs are rat-specific. Given that only one rat miRNA was added from the miRbase version 16 to the latest version 18, discovery of 14 novel rat miRNAs will significantly contribute to the understanding of miRNA in rat gene expression.

## Materials and Methods

### Ethical Treatment of Animals

National Center for Toxicological Research (NCTR) Institutional Animal Care and Use Committee (IACUC) reviewed and approved this study. We followed the recommendations of the NCTR IACUC for the handling, maintenance, treatment and sacrifice of the rats. All efforts were made to minimize the animal suffering.

### miRNA Isolation

Aristolochic acid (AA) was purchased from Sigma (St. Louis, MO). The purity of AA was 96% (40% of AAI and 56% of AAII). Big Blue transgenic Fisher 344 rats were obtained from Taconic Laboratories (Germantown, NY) through a purchase from Stratagene (La Jolla, CA). The miRNA isolation from four AA-treated and 4 control rats [56] was performed as previously described [45]. Briefly, 40–50 mg rat kidney was cut and mechanically minced using Tissue Tearor (Biospec Products Inc, Bartlesville, OK). Total RNA was isolated using mirVana™ miRNA isolcation kit (Ambion, TX) that employed an organic extraction followed by glass-fiber immobilization. RNA concentration was determined using Nandrop1000 spectrophotometer (Thermo Scientific, DE). The quality of the extracted RNA was evaluated using the RNA 6000 LabChip and Agilent 2100 Bioanalyzer (Agilent Technologies, Palo Alto, CA).

### Small RNA Library Construction

The small RNA library construction and deep sequencing was carried out at University of Texas Southwestern Medical Center Microarray Core Facility. Samples were prepared using Illumina Small RNA Sample Prep kit according to the Small RNA v1.5 Sample Preparation Guide. Approximately 10 µg of total RNA was used for the small RNA library construction. The v1.5 sRNA 3′ and SRA 5′ adaptors (Illumina, San Diego, CA, USA) were added to both ends of the small RNAs. The 3′ and 5′ ligated RNAs were used as templates for reverse transcription followed by PCR amplification. The enriched cDNA constructs were size-fractionated on a 6% polyacrylamide gel electrophoresis and the bands containing the 22–30 nucleotide RNA fragments (93–100 nucleotide in length with both adapters) were purified. The concentrations of the size-fractionated cDNA libraries were determined using a NanoDrop ND-1000 Spectrophotometer and the size and purity were determined using an Agilent 2100 Bioanalyzer in combination with the Agilent DNA 1000 Kit. The purified DNA was used directly for cluster generation and sequence analysis using the Illumina Genome Analyzer II (Illumina) according to the manufacturer's instructions (36 cycle single read cluster kit v4 and sequence kit v4). Images taken during the sequencing reactions were analyzed with the Illumina software, performing the base-calling with Bustard and sequence analysis with Gerald.

### Identification of Novel miRNA Candidates

To predict novel miRNAs, the miRanalyzer standalone version [34] was used. All reads that were mapped to a non-redundant set of known rat miRNAs from miRBase version 17 were removed. All mappings are performed using Bowtie, an ultrafast and memory-efficient alignment program for aligning short DNA sequence reads to genomes [57]. The remaining reads were then aligned to a non-redundant set of all known miRNAs except for rat miRNAs from miRBase version 17. These mapped reads were retained and considered as belonging to putatively homologous miRNAs (detected in other species but so far not in rat). Those retained reads were mapped to the rat genome with a seed length of 19 nt allowing 1 mismatch. The genome-mapped reads were then clustered on the rat genome and the read clusters were used for the prediction of miRNAs as described previously [34]. Thus, the novel miRNAs detected in this way are homologous to those in other species.

To detect rat-specific novel miRNAs, all reads that were mapped to known miRNAs in miRBase version 17 and other known small RNAs were removed. The known small RNAs include 1) RNA from RFam 10.1 [58], 2) tRNA from the GtRNAdb [59] and 3) piRNA from RNAdb [60] and mRNAs from The Reference Sequence (RefSeq) database [61]. The remaining reads were input into miRanalyzer for analysis to select the candidate rat-specific novel miRNAs.

The consensus sequences of the novel rat homologous, rat-specific mature and pre-miRNAs were predicted at least 4 of 8 rat kidney samples by the miRanalyzer differential expression module. The NGS reads from all 8 samples were then mapped to the rat genome. Novel rat pre-miRNAs were identified based on the presence of a classic hairpin structure, Dicer cleavage pattern (a characteristic 2 nucleotide 3′ overhang), the mature and mature* sequences, and conservative 5′ sequence, as well as detectable expression (NGS read count).

Mature miRNAs tend to have several length variants and the consensus sequence frequently is found to be longer than the predominant form (the most expressed read) [10]. Here, the length of the most expressed read was considered as the length of the mature miRNAs. The pre-miRNA is defined as the sequence that starts at the first bulge (regions in which one strand of a miRNA has “extra” inserted bases with no counterparts in the opposite strand) before the 5′ mature miRNA and ends at the corresponding position in 3′. The minimum length of pre-miRNA is 65 nt if the flanking side of the pre-miRNA does not reach the next bulge.

The secondary structures of rat miRNAs were determined by the RNAfold using minimum free energy (MFE) algorithm. RNAfold is a web server and widely used for prediction of RNA structures [62], [63].

### Custom Vertebrate miRNA Microarray

Microarray assay was performed using a service provider (LC Sciences, Houston, TX). The assay started from 4 to 8 µg total RNA per sample. The RNA was 3′-extended with a poly (A) tail using poly (A) polymerase. An oligonucleotide tag was then ligated to the poly (A) tail for later fluorescent dye staining. Hybridization was performed overnight on a µParaflo microfluidic chip using a micro-circulation pump (Atactic Technologies, Houston, TX) [64], [65]. Hybridization used 100 µL 6×SSPE buffer (0.9 M NaCl, 60 mM Na_2_HPO_4_, 6 mM EDTA, pH 6.8) containing 25% formamide at 34°C. After RNA hybridization, tag-conjugating Cy3 dye was circulated through the microfluidic chip for dye staining. Fluorescence images were collected using a laser scanner (GenePix 4000B, Molecular Device, Sunnyvale, CA) and digitized using Array-Pro image analysis software (Media Cybernetics, Bethesda, Maryland). Data were analyzed by first subtracting the background and then normalizing the signals using a LOWESS filter (Locally-weighted Regression) [66]. Data adjustment included data filtering, Log2 transformation, and gene centering and normalization. The data filtering removed miRNAs with intensity values below a threshold value of 32 across all samples. T-test was performed between “control” and “test” sample groups to determine the *p*-value [67].

### Prediction of miRNAs' Target Genes

TargetSpy, an algorithm for prediction of miRNA target genes, was used to predict the target genes of the nine novel rat miRNAs [48], The principle of prediction of miRNA target genes is based on machine learning and selected features, such as compositional, structural, and base pairing features (http://www.targetspy.org/). TargetSpy has been demonstrated to have good prediction accuracy and is used to predict miRNAs targets genes [68].

## Supporting Information

Data S1 **Predicted target genes of the fourteen rat novel miRNAs.**
(XLS)
